# QuaDMutEx: quadratic driver mutation explorer

**DOI:** 10.1186/s12859-017-1869-4

**Published:** 2017-10-24

**Authors:** Yahya Bokhari, Tomasz Arodz

**Affiliations:** 1 Department of Computer Science, School of Engineering, Virginia Commonwealth University, 401 W. Main St., Richmond, 23284 VA USA; 2 Center for the Study of Biological Complexity, Virginia Commonwealth University, Richmond, 23284 VA USA

**Keywords:** Somatic mutations, Cancer pathways, Driver mutations

## Abstract

**Background:**

Somatic mutations accumulate in human cells throughout life. Some may have no adverse consequences, but some of them may lead to cancer. A cancer genome is typically unstable, and thus more mutations can accumulate in the DNA of cancer cells. An ongoing problem is to figure out which mutations are drivers - play a role in oncogenesis, and which are passengers - do not play a role. One way of addressing this question is through inspection of somatic mutations in DNA of cancer samples from a cohort of patients and detection of patterns that differentiate driver from passenger mutations.

**Results:**

We propose QuaDMutEx, a method that incorporates three novel elements: a new gene set penalty that includes non-linear penalization of multiple mutations in putative sets of driver genes, an ability to adjust the method to handle slow- and fast-evolving tumors, and a computationally efficient method for finding gene sets that minimize the penalty, through a combination of heuristic Monte Carlo optimization and exact binary quadratic programming. Compared to existing methods, the proposed algorithm finds sets of putative driver genes that show higher coverage and lower excess coverage in eight sets of cancer samples coming from brain, ovarian, lung, and breast tumors.

**Conclusions:**

Superior ability to improve on both coverage and excess coverage on different types of cancer shows that QuaDMutEx is a tool that should be part of a state-of-the-art toolbox in the driver gene discovery pipeline. It can detect genes harboring rare driver mutations that may be missed by existing methods. QuaDMutEx is available for download from https://github.com/bokhariy/QuaDMutEx under the GNU GPLv3 license.

## Background

Cancer is a complex and heterogeneous disease that starts at cellular level as a consequence of a hereditary or, most prevalently, environmentally induced mutations [1, 2]. Mutations such as amino acid substitutions or copy number alterations may lead to abnormal cells that can divide indefinitely and have the ability to invade other tissues [3]. A sequence of between two and eight mutations that target genes involved in specific cell functions is needed for most human cancers to develop [4]. Such mutations, which confer growth advantage to cells and are causally implicated in oncogenesis, are referred to as driver mutations [5]. Known somatic mutations linked to cancer, often with additional information such as known therapies that target the mutation, are being gather in databases [6–8] that can be used in selecting patient treatment. Newly identified driver genes can also be screened using druggability indices [9], and for being targets for drug repositioning [10], leading the way to new therapeutic modalities. Thus, discovering and cataloging genes whose mutations do contribute to oncogenesis, that is, driver genes, is a major goal for experimental and computational cancer research. The wide spectrum of approaches for finding driver genes can be seen in recent review papers [11–13].

The ability to discover driver mutations has been moved forward in recent years owing to the availability of large datasets generated using second-generation sequencing techniques [14]. Projects such as the Cancer Genome Atlas (TCGA) [15] perform sequencing of matched tumor and normal samples from hundreds of patients with a given tumor type, allowing for detection of somatic mutations present in tumor tissue. However, even with the increasing availability of data, the problem of identifying driver mutations and driver genes that harbor them is far from being solved.

The main challenge is that majority of somatic mutations acquired in human cells throughout life are not causally linked to cancer. It is estimated that a typical human cell, with a genome consisting of approximately 3×10^9^ base pairs, gains on the order of 10^−10^ mutations per base pair per cell division [16, 17], although the rate can vary substantially depending on factors such as local chromatin organization of the genome [18]. Human organism consists of on the order of 10^13^ cells [19], many of which are in fast dividing tissues; for example around 10^11^ epithelial cells are being lost and need replacement every day [20]. It is thus evident that most mutations do not lead to carcinogenesis - these are often referred to as passenger mutations. Indeed, it has been observed that in tissues that self-renew through cell division, such as skin or gastrointestinal epithelium, the number of mutations seen in cancer samples from patients 85 years old is twice the number of mutations in patients that are 25 years old. It has been estimated that half or more of all mutations observed in patients’ cancer tissues originate prior to the onset of cancer [17]. In addition to these mutations, cancer cells exhibit a mutator phenotype, that is, an increased mutation rate [21], with mutation rates that can differ by an order of magnitude among subclones within the tumor [22]. This further contributes to the dominance of passenger mutations over driver mutations in observed cancer tissue samples. Altogether, while the number of driver mutations in a tumor is typically small – a recent analysis of TCGA data shows it to be between 2 and 6 in most tumors [23] – the total number of somatic mutations present in a single patient tumor tissue sample can range between 10 to above 100, depending on tissue type and patient age. Most mutations in a cancer tissue sample are thus passenger mutations that do not contribute positively to cancer growth. In fact, weakly deleterious effects of multiple passenger mutations can accumulate and can have negative impact on the tumor [24].

To discover driver mutations in the abundance of passenger mutations, many approaches take the route of calculating the background mutations rate that would be exhibited by passenger mutations, and consider those mutations that are observed more frequently as drivers. These approaches employ a statistical model of somatic mutations, typically considering them a result of a Poisson process, which allows for quantifying the statistical significance of any deviations from the background mutation rate. For example, MutSig [25] uses a constant mutation rate across all genes, and can also use methods for functional predictions of mutation significance, such as SIFT [26], CHASM [27], Polyphen-2 [28] and MutationAssessor [29]. MutSigCV [30] uses factors such as chromatin state and transcription activity to estimate gene-specific background mutation rates. PathScan [31] utlizes a Poissonian mutation model that involves gene lengths, and for a gene set given by the user calculates the probability of observing that many mutations or more under a null hypothesis that the mutations are passengers. If the probability is low across many samples, the genes are considered driver genes. MuSiC [32] extends PathScan by adding knowledge about correlation between mutation rates and factors including clinical variables such as age, molecular variables such as the Pfam family to which the genes belong, and sequence correlates such as base composition of the site and proximity among mutation sites. DrGaP tool [33] considers 11 different types of mutation types, with factors including G/C content near the mutation site and methylation status of the site, in estimating the background mutation rate. DOTS-Finder [34] integrates functional predictions and background mutation rate to identify driver genes.

Gene-centric methods for finding driver mutations from cancer sequencing data are hampered by the fact that a single driver gene is rarely mutated across many patients with a given tumor. Only few genes, such as TP53 or BRCA1, are mutated in large fraction of cases. Most driver mutations are relatively rare in tumor patients: most of individual genes are mutated in less than 5% of patients [35]. Thus, a statistically significant detection of deviation from background mutation rate requires large number of samples for rare drivers.

Observations from cancer samples show the disease-linked mutations are not confined to a specific set of loci but, instead, they differ substantially in individual cases. Only when seen from the level of pathways, that is, genes related to a specific cellular process, a clearer picture emerges. A study of pancreatic cancer has identified a core of altered pathways common to all cases, and additional variant pathways [36] altered in some of the patients. This evidence has given rise to network-oriented driver detection methods, such as HotNet [37, 38], which incorporates protein-protein networks and uses a heat diffusion process, in addition to gene mutation frequency, to detect a driver subnetwork. Some methods move beyond utilizing mutation data. For example, MEMo [39] uses gene expression to filter out genes with copy number alterations that do not show altered expression. A more refined way of incorporating gene expression data is used by DriverNet [40], which analyzes if a mutation in a gene affects expression of genes it regulates.

In many types of tumors, only one mutation per pathway, or functionally related group of genes, is needed to drive oncogenesis [41–43]. Thus, the minimal set of mutated genes required for cancer to develop would consists of several sets of genes, each corresponding to a crucial pathway such as angiogenesis. Within each gene set, in each patient exactly one gene would be mutated. That is, all patients would be covered by a mutation in a gene from the set, and there would be no excess coverage, that is, no patient will have more mutations than one in the genes from the set. This pattern has been often referred to as mutual exclusivity within a gene set, and several methods, including Dendrix [44] and Multi-Dendrix [45], RME [46], CoMEt [47], TiMEx [48] and MEMo [39] detect set of driver genes by quantifying mutual exclusivity. Further methods extend these by helping deal with observation errors in the data [49], and with computational efficiency of the search for driver genes [50].

Mutual exclusivity describes the combinatorial pattern of a minimal set of genes required for oncogenesis. In actual patient data, additional mutations in driver genes may occur, especially for slow growing tumors. Also, some of the mutations may be missed due to observation errors. Thus, instead of detecting the presence or absence of mutual exclusivity in a set of genes, driver detection algorithms involve a score that penalizes for deviations from a driver pattern. That is, a penalty is incurred for zero mutations in a patient, or for more than one mutation. Then, a heuristic search procedure is utilized to find a set of genes closest to the mutual exclusivity pattern, since finding such a set has been shown to be an NP-hard problem [44].

Here, we propose a tool, QuaDMutEx, which brings three novel aspects to the mutual-exclusivity-based driver detection. First, instead of linear penalty for excess coverage used in tools like Dendrix, QuaDMutEx uses a quadratic penalty that provides a more realistic penalty for sets with excessive number of mutations. Second, the method allows for user-specified trade-off between increasing coverage and decreasing excess coverage, allowing for tailoring the method to fast- or slow-evolving tumors. Third, QuaDMutEx uses a combination of optimal search that results in globally optimal solutions to subproblems with a stochastic search through a series of subproblems, allowing for more effective search through the space of possible driver gene sets. We evaluated our method on data obtained literature and from the Cancer Genome Atlas. Our method shows higher coverage and higher mutual exclusivity than four state-of-the-art tools: Dendrix, TiMEx, RME and CoMEt. Compared to DriverNet, a non-exclusivity based tool, it returns complementary sets of putative cancer driver genes of comparable quality when evaluated against the COSMIC cancer database.

## Methods

The proposed algorithm for detecting driver mutations in cancer operates at the gene level. That is, on input, we are given an *n* by *p* mutation matrix *G*, where *n* is the number of cancer patients with sequenced cancer cell DNA, and *p* is the total number of genes explored. The matrix is binary, that is, *G*_*ij*_=1 if patient *i* has a non-silent mutation in gene *j*; otherwise, *G*_*ij*_=0. A row vector *G*_*i*_ represents a row of the matrix corresponding to patient *i*. The solution we seek is a sparse binary vector *x* of length *p*, with *x*_*j*_=1 indicating that mutations of gene *j* are cancer driver mutations. We will often refer to the nonzero elements of *x* as the mutations present in *x*.

In designing the algorithm for choosing the solution vector *x*, we assumed that any possible vector is penalized with a penalty score based on observed patterns of driver mutations in human cancers. We expect that each patient has at least one mutation in the set of genes selected in the solution; however, in some cases, the mutation may not be detected. Also, while several distinct pathways need to be mutated to result in a growing tumor, typically one mutation in each of those pathways suffices. The chances of accumulating additional mutations in the already mutated pathway are low, and decrease with each additional mutation. We capture this decreasing odds through a quadratic penalty associated with *x* given the observed mutations *G*_*i*_ in patient *i*

1 $$\begin{array}{*{20}l} L(G_{i},x)=\frac{1+k}{2}\left(G_{i} x - 1\right)\left(G_{i} x - \frac{2}{1+k}\right). \end{array} $$

**Fig. 1:**
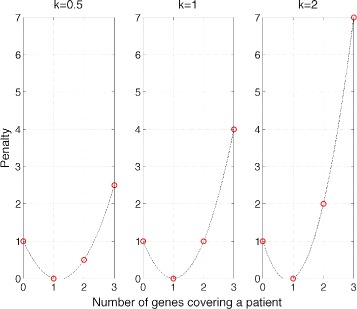
Effect of different values of parameter *k* on penalty *L*(*G*_*i*_,*x*), in a function of *G*_*i*_ *x*, i.e., the number of mutations from solution *x* present in patient *i*

The term *G*_*i*_ *x* captures the number of mutations from solution *x* present in patient *i*. The penalty is parameterized by a non-negative real number *k* to be chosen by the user. It captures the ratio of penalty for exactly two mutations in genes from set *x* present in patient *i* to penalty for no mutation from set *x* present in patient *i*. We incur no penalty if the number of mutated genes from *x* in a given patient is one. The effect of *k* on the penalty can be seen in Fig. 1. For example, for a tumor with strong mutator phenotype where more mutations are present one can set *k* to a low value, lowering the penalty for multiple mutations in genes from set *x* present in a patient.

**Fig. 2:**
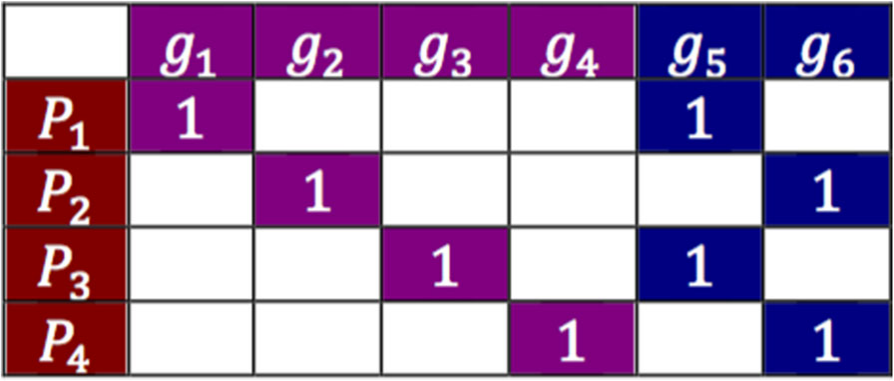
Illustration of the role of the penalty for the solution size on the driver selection problem with six genes and four patients. Without the *L*_0_ term, either violet or blue genes are equally good optimal solutions. Inclusion of *L*_0_ pseudo-norm makes the blue solution a preferred one

In addition, we expect that the number of genes harboring driver mutations in a given pathway is small. Hence, we introduce a penalty on the number of genes selected in the solution, in a form of *L*_0_ pseudo-norm, *L*_0_(*x*)=||*x*||_0_. The effect of introducing the penalty can be seen in Fig. 2.

The total penalty for a possible solution vector *x* is a sum of per-patient penalties and the solution-size penalty:

2 $$\begin{array}{*{20}l}{} L(G,x)&=\sum\limits_{i=1}^{n} L(G_{i},x) + C L_{0}(x)\\ &= \sum\limits_{i=1}^{n} \frac{1+k}{2}\left(G_{i} x - 1\right)\left(G_{i} x - \frac{2}{1+k}\right) + C ||x||_{0}. \end{array} $$

The parameter *C* controls the trade-off between minimization of *L*(*G*_*i*_,*x*) terms and of the *L*_0_ pseudo-norm. It can alternatively be seen as the penalty incurred by increasing the number of genes in the solution *x* by one.

Minimization of *L*(*G*,*x*) can be viewed as an unconstrained binary quadratic problem (BQP) with the solution space involving binary vectors *x* of length *p*:

3 $$\begin{array}{*{20}l} & \underset{x}{\text{minimize}} & & {x^{T}Qx}-f^{T}x  \\ & \text{subject to} & & 0\leq x\leq1 \\ &&& x \in \mathbb{Z} \\ & \text{where} & & Q= \frac{k+1}{2} G^{T}G \\ & & & f=\frac{k+3}{2}G^{T}\mathbf{1}_{n}- C\mathbf{1}_{p}  \end{array} $$

where **1**_*n*_ represents a unit vector of length *n*.

BQPs are known to be NP-hard in general [51]. However, the optimal solution can be obtained quickly for problems of small size. Our approach in solving this problem involves a meta-heuristic based on Markov-Chain-Monte-Carlo search combined with optimal local search for small subproblems. The algorithm is presented below.

The main QuaDMutEx algorithm goes through *T* iterations, and in each considers a solution *x* containing up to *ν* genes. In each iteration, a new candidate solution is generated by randomly modifying the current solution vector. The new candidate solution is then modified by dropping some genes, based on exact binary quadratic optimization (Eq. 3) involving *ν* genes present in the candidate solution. If the optimized solution is better than the solution from previous iteration, it is accepted. If not, it is accepted with probability depending on the difference in quality of the previous and the current solution. Throughout iterations, the solution *x* ^∗^ with the lowest value of the objective function (Eq. 2) is kept.



The random process generating a new candidate solution based on current solution always returns a solution with exactly *ν* genes. If the current solution already has *ν* genes, one of them will be randomly replaced with a gene not in the solution. The gene to be removed is chosen at random with uniform probability of 1/*ν*. The gene to be added is chosen by random sampling from a distribution *Γ*_∼*x*_, which is defined through a user-supplied distribution *Γ* over all genes, modified to have 0 probability for the genes currently in solution *x*. If the current solution contains less than *ν* genes, the solution is expanded to include *ν* genes, and the *ν*−||*x*||_0_ genes to be added are sampled without replacement according to *Γ*_∼*x*_. In our experiments, we used *Γ* proportional to the logarithm of the frequency of a mutation in a given gene among patients in the dataset.



The local search for an improved new solution returns an optimized solution *x* and its penalty score, *L*. It operates by limiting the problem to the *ν* genes present in the new candidate solution. That is, we create a *n* by *ν* submatrix *G*_*x*_ by choosing from *G* columns for which *x*=1. Thus, we have an NP-hard binary QP problem with number of variables small enough that that problem can be quickly solved to the optimum using standard techniques. In our experiments, for datasets with below 1000 patients, values of *ν* up to 50 lead to BQP problems where global optimum could be reached in less than a second on a desktop workstation.



In the proposed approach, the solution vector *x* from a single run of QuaDMutEx will capture a set of driver genes that are functionally related and thus exhibit mutual exclusivity pattern, for example genes that are all part of a pathway that needs to be mutated in oncogenesis. To uncover a comprehensive set of driver genes for a specific cancer type, spanning multiple functional subsystems vital to oncogenesis, the algorithm should be applied multiple times, each time removing the genes found in prior runs from consideration.

## Results and discussion

### Evaluation on real cancer datasets

We evaluated the proposed algorithm using four somatic mutation datasets (see Table 1), one from the Cancer Genome Atlas (TCGA) database and three from literature. Two datasets were originally used by the authors of Dendrix: somatic mutations in lung cancer (LUNG), and a dataset relating to Glioblastoma Multiforme (GBM) that includes not only somatic mutations but also copy number alternations. The ovarian cancer dataset (OV) was originally used by the authors of TiMEx tool [48]. A larger dataset of mutations in samples from Breast Invasive Carcinoma (BRCA) was downloaded from TCGA. Following standard practice, in the BRCA dataset we removed known hypermutated genes that have no role in cancer [30], including olfactory receptors, mucins, and a few other genes such as titin. For each dataset, each gene in each patient was marked with one if it harbored one or more mutation, and with zero otherwise, resulting in the input matrix *G* for QuaDMutEx.

**Table 1:** Summary of mutation-only datasets used in experimental validation of QuaDMutEx

Dataset	Samples (n)	Genes (p)	Mutations
GBM	84	178	809
OV	316	312	3004
LUNG	163	356	979
BRCA	771	13,582	33,385

### Quantitative evaluation of QuaDMutEx results

We ran QuaDMutEx on the four datasets: GBM, OV, LUNG, and BRCA. In the tests, we set the maximum size of the gene set to be *ν*=30. We set *k*=1, indicating neutral stance with respect to the trade-off between coverage and excess coverage. The value of *C*, the weight of the gene solution size penalty, was set to 0.5 for GBM, the dataset with the smallest number of genes measured, to 1 for the LUNG and OV datasets which have twice the number of genes compared to GBM, and to 1.5 for BRCA, the dataset with much larger number of genes. We ran QuaDMutEx for 10,000 iterations, which corresponds to running times below 10 minutes for each dataset. For GBM and BRCA, we also ran additional experiments with the default parameter values: *k*=*C*=1.

To assess statistical significance of the results returned by QuaDMutEx, we used the method proposed in [44]. In short, we randomly permuted the contents of each column of the input patient-gene matrix, which results in randomized dataset in which, for each gene, the number of patients harboring a mutation in the gene is preserved, but any pattern of mutation within a row, that is, within each single patient, is lost. We created 1000 randomized datasets and ran QuaDMutEx on each dataset. The value of the objective function observed on the original dataset was then compared with the distribution of objective function values on the randomized datasets to obtain a *p*-value estimate. The results of the tests, presented in Table 2, show that for all four datasets, QuaDMutEx returns gene sets that are statistically significant at 0.05.

**Table 2:** Quantitative characteristics of QuaDMutEx results. For all four datasets, the solutions are statistically significant at *p*<0.05

Dataset	Parameters	Genes	Quadratic	Estimated
			penalty	*p*-value
GBM	*k*=1,*C*=0.5	12	18	0.023
GBM	*k*=*C*=1 (default)	7	20.5	0.001
OV	*k*=*C*=1 (default)	3	17	0.010
LUNG	*k*=*C*=1 (default)	15	59	0.036
BRCA	*k*=1,*C*=1.5	20	393	0.002
BRCA	*k*=*C*=1 (default)	26	399	0.002

The quadratic penalty provides a single-metric measure for what is essentially a two-criterion optimization problem involving simultaneous maximization of coverage and mutual exclusivity. To capture each of these independently, we used two metrics, coverage and excess coverage:

- *coverage* = $\frac {\text {number of patients covered by at least one gene from the set}}{\text {total number of patients}}$
- *excess coverage*= $\frac {\text {number of patients covered by more than one gene from the set}}{\text {number of patients covered by at least one gene from the set}}$

These metrics together capture how well a gene set conforms to the pattern expected of driver genes. Both of the metrics range from 0 to 1. A perfect pattern would have coverage of 1 and excess coverage of 0, indicating full mutual exclusivity.

### Comparison with other mutual-exclusivity-based methods

For comparison, we used RME [46], TiMEx [48], CoMEt [47] and Dendrix [44] as they are all from the de novo discovery family of methods [11] for driver detection, and all utilize only genetic data, same as QuaDMutEx. We ran the four tools on the same four datasets: GBM, OV, LUNG, and BRCA. For TiMEx,, which does not require the user to specify the number of genes in the solution, we ran the tool with default parameters. Dendrix, RME and CoMEt require the user to provide the desired solution size. For Dendrix, we performed 29 runs for each dataset, with the solution size parameter ranging from 2 genes to 30 genes, and picked the solution size with the best Dendrix score. Each run involved 10^7^ iterations. For CoMEt the running time increases steeply with the requested solution size, thus we used sizes for which a single run finishes in less than 48 h; in result, we tested solution sizes 2, 3, 4,5 for GBM and OV, between 2 and 6 for LUNG, and between 2 and 10 for BRCA. For RME, we used solution sizes between 2 and 5 genes, as recommended by the authors of the tool. For BRCA dataset, RME invoked with default parameters does not return any valid solution; to circumvent this problem, we executed RME for BRCA with the minimum gene frequency parameter lowered to 0.02 from the default value of 0.1. For the other three datasets, we used the default value.

We used the objective function maximized by Dendrix, which can be expressed using the notation introduced in the Methods section as *Dendrix score* = $n - \sum _{i=1}^{n} |G_{i} x - 1|$, as the metric for evaluating the tool. Essentially, the Dendrix score equals to total coverage minus coverage overlap, where total coverage is the number of patients covered by at least one gene from the given gene set, and coverage overlap is total count of all mutations in genes from the set that are in excess of one mutation per patient. High-quality solutions should have high Dendrix score.

**Fig. 3:**
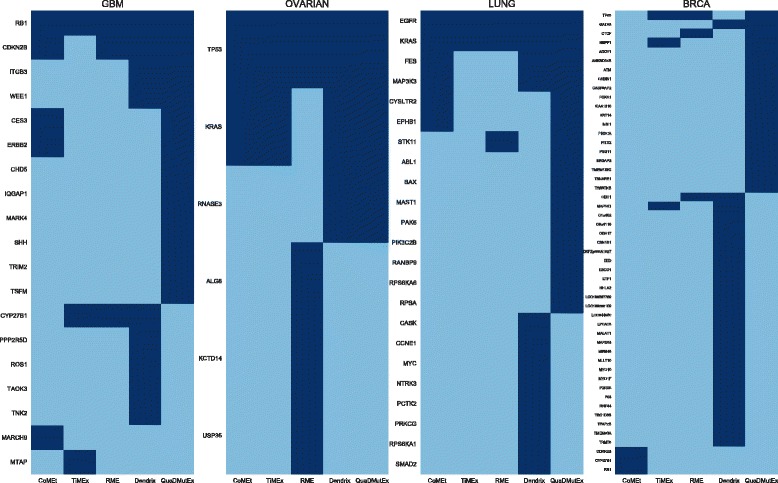
Comparison of putative cancer driver gene sets returned by QuaDMutEx and the other tools. Genes found by a tool are in dark blue

The results of the tests, presented in Table 3, show that QuaDMutEx consistently returns higher quality solutions than all other methods. Only on the OV dataset, Dendrix discovers the same set of genes as QuaDMutEx. Remarkably, the quality of solutions from QuaDMutEx is higher even though the score used as the metric, the Dendrix score, is not function optimized by QuaDMutEx, but is the objective function of Dendrix. These results show that the proposed optimization scheme that combines stochastic heuristic approach with exact solution to a series of tractable subproblems is more efficient than the heuristic approach employed in Dendrix. The putative cancer driver gene sets discovered by QuaDMutEx are mostly different than sets returned by other tools (see Fig. 3).

**Table 3:** Comparison between QuaDMutEx and other methods. For QuaDMutEx, we used default parameter values *k*=1 and *C*=1 unless specified otherwise

Method	Genes	Coverage	Excess	Dendrix
			coverage	score
GBM: Glioblastoma multiforme				
TiMEx	3	0.7857	0.0606	62
RME	3	0.7857	0.0606	62
CoMEt	5	0.8452	0.0845	65
Dendrix	9	0.8571	0.0556	68
QuaDMutEx (C=0.5)	12	0.9286	0.0769	*72*
QuaDMutEx	7	0.8690	0.0822	67
OV: Ovarian Cancer				
TiMEx	2	0.9525	0	301
RME	5	0.9494	0.1	62
CoMEt	2	0.9525	0	301
Dendrix	3	0.9557	0	*302*
QuaDMutEx	3	0.9557	0	*302*
LUNG: Lung Adenocarcinoma				
TiMEx	2	0.5521	0	90
RME	3	0.6748	0.1273	96
CoMEt	6	0.6196	0	101
Dendrix	12	0.6809	0.0270	108
QuaDMutEx	15	0.8160	0.1053	*119*
BRCA: Breast Invasive Carcinoma				
TiMEx	3	0.4202	0.1006	289
RME	3	0.3865	0.0268	290
CoMEt	3	0.2620	0	202
Dendrix	29	0.5811	0.09598	402
QuaDMutEx (C=1.5)	20	0.6109	0.1338	408
QuaDMutEx	26	0.6342	0.1595	*411*

Highest result for each dataset indicated in italics

**Fig. 4:**
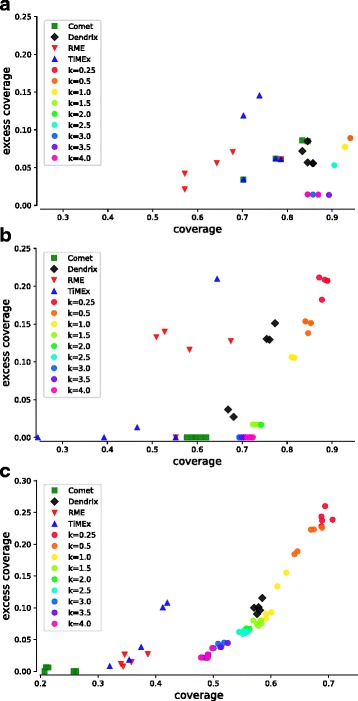
Comparison of results from QuaDMutEx using different values of parameter *k* with results from other tools, in terms of coverage and excess coverage: **a**) GBM; **b**) LUNG; **c**) BRCA. In all three datasets, QuaDMutEx results are on the Pareto frontier

We also checked how QuaDMutEx performs with respect to coverage and excess coverage, and compared the results with those of Dendrix, RME, TiMEx, and CoMEt. One of the features of QuaDMutEx is the flexibility in choosing the parameter *k*, which controls the trade-off between high coverage but higher excess coverage solutions and low excess coverage but lower coverage solutions. Thus, we ran QuaDMutEx with a range of values of parameter *k*=0.25,0.5,1,1.5,2,2.5,4. As previously, the value of *C* was set to 0.5 for GBM, to 1 for the LUNG and OV, and to 1.5 for BRCA. The number of iterations was again set to 10,000. For each parameter setting, we ran QuaDMutEx 5 times. We also gathered results from 5 runs of Dendrix for the best-performing solution size. For RME, TiMEx, and CoMEt the results do not vary from run to run, so we instead picked top five solution from a single run. Then, we quantified coverage and excess coverage. The results in Fig. 4 show that QuaDMutEx solutions are on the Pareto-optimality frontier of all (RME, TiMEx, CoMEt, Dendrix and QuaDMutEx) solutions. For each Dendrix, TiMEx and CoMEt solution, there is a QuaDMutEx solution that is better: has higher coverage and lower excess coverage. These results further confirm results from Table 3 showing that the proposed tool improves upon the state-of-the-art. Data for OV are not shown graphically, as there is very little variability in solutions returned by the methods and the plot only confirms what is presented in Table 3.

### Effects of parameters on QuaDMutEx

The proposed methods allows for adjusting the penalty for expanding the solution size, through a parameter *C* that corresponds to the additional penalty for increasing the number of genes in the solution by one. It also allows for tweaking the trade-off between coverage and mutual exclusivity, through a parameter *k* that captures the ratio of penalty for one excess mutation in a patient to penalty for the patient not being covered by any mutation. We have analyzed how these two parameters affect the solution by running QuaDMutEx for 10,000 iterations for parameters *C*=0.25,0.5,1,1.5,2,2.5,4 and *k*=0.25,0.5,1,1.5,2,2.5,4.

**Fig. 5:**
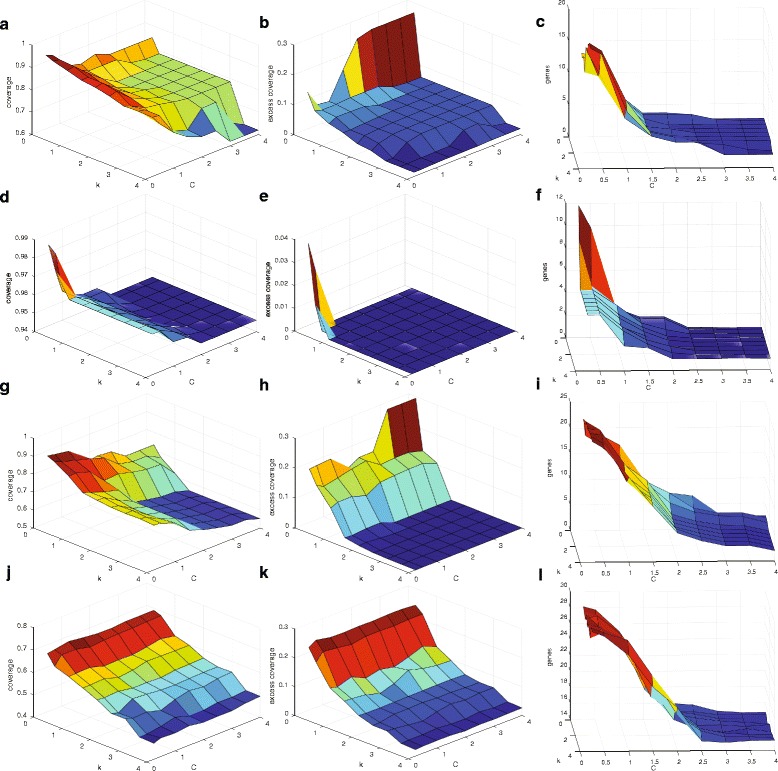
Effects of parameters *C* and *k* on QuaDMutEx results, i.e., coverage (**a**,**d**,**g**,**j**), excess coverage (**b**,**e**,**h**,**k**), and genes in solution (**c**,**f**,**i**,**l**), for GBM dataset (**a**,**b**,**c**), OV dataset (**d**,**e**,**f**), LUNG dataset (**g**,**h**,**i**), and BRCA dataset (**j**,**k**,**l**)

Figure 5 shows that the parameter *C* achieves its design goal, that is, solutions with higher *C* include fewer genes. The figures also show that as the penalty for the size of the solution set is lowered, by specifying lower value of *C*, the coverage of patients by genes in the solution tends to increase for the three small datasets, where high values of *C* reduce the solution size to only a few genes and thus necessarily lower coverage. This effect is not present in the large dataset, BRCA, where *C* does not impact coverage. Changing *C* does not show any impact on excess coverage.

Changes in parameter *k* result in changes in coverage and excess coverage, but has no substantial impact on the number of genes in the solution. The results show that, as intended, lower values of *k* lead to higher coverage, at the cost of higher excess coverage, than high values of *k*. Thus, for slow growing tumors, tumors with elevated mutator phenotypes, or tumors in old patients, where many mutations may occur by chance and higher excess coverage is expected, low values of *k* is preferred over high *k* values.

### Qualitative assessment of QuaDMutEx results

To validate the ability of QuaDMutEx to take only mutation data and discover rare putative cancer driver genes, which are the most hard to find using traditional methods that rely on mutation frequency in patient population, in each of the four datasets we focused on the genes in the solution with the fewest number of mutations. See Table 4 for a complete list of all genes in the solution, and for the number of mutations for each gene in each dataset. In addition to literature review, we also used DriverDBv2 [7], a database of previously discovered cancer driver genes, to further validate the quality of QuaDMutEx solutions.

**Table 4:** Putative driver gene sets discovered by QuaDMutEx

Putative driver genes	Estimated
discovered by QuaDMutEx	*p*-value
GBM: Glioblastoma multiforme	
**CDKN2B** (43) **TSFM** (16) **RB1** (10) **ERBB2** (7) **ITGB3** **TRIM2** **WEE1** **CHD5 MARK4 CES3 SHH IQGAP1** (1)	0.023
OV: Ovarian cancer	
**TP53** (299) **KRAS** (2) RNASE3 (1)	0.010
LUNG: Lung Adenocarcinoma	
**KRAS** (60) **STK11** (34) **EGFR** (30) **EPHB1** (4) **MAP3K3** (3) **ABL1 PAK6 MAST1 CYSLTR2 RPS6KA6** **FES** (2) **BAX PIK3C2B RANBP9** RPSA (1)	0.036
BRCA: Breast Invasive Carcinoma	
**TP53** (194) **PIK3CA** (138) **GATA3** (80) NBPF1 (27) **CTCF** (18) **ATM** (16) **FOXA1** (15) **TMEM132C** (6) **CABIN1** **SRGAP2** KIAA1310 (5) **CASP8AP2** **TSNARE1** (4) **ADCY1 PITX2 PSG11** (3) **ANKRD34B KRT14 MSI1 TWISTNB** (2)	0.002

For each gene, in parentheses, we provide the number of patients in the dataset that harbored a mutation in that gene. Genes in bold are present int the DriverDBv2 [7] database of previously discovered cancer drivers

In the brain tumor dataset, eight identified genes are each mutated in only 1 out of 84 patients. Out of these, ITGB3 has known role in multiple cancers [52, 53], TRIM2 has tumor suppressing function in ovarian cancer [54] and plays a role in brain, the source of the analyzed tissue [55], WEE1 is already a target for cancer therapy [56], and CHD5 is a known tumor suppressor [57]. Changes in expression of MARK4 have been observed in glioblastomas [58]. While no cancer role has been so far identified for carboxylesterase 3 (CES3), it is known to be expressed in the source tissue of our samples, the brain [59]. SHH gene has been linked to glioma growth [60], as well as to other cancers [61]. Finally, IQGAP1 is believed to play a role in cell proliferation and cancer transformation [62].

In the ovarian cancer dataset, KRAS, a known proto-oncogene, was found mutated in two patient. Eosinophil cationic protein (RNase 3) was found in only one patient. The protein, while not present in DriverDBv2 and not directly related to oncogenesis, has cytotoxic activity and was recently shown to inversely affect viability of cancer cell lines [63] and thus its mutations may affect human tumor growth.

In the QuaDMutEx solution for the lung datasets, six putative cancer driver genes are each mutated in only two of the 356 patients, and additional four are mutated in single patients. Among these, role of ABL1 in cancer is well established. PAK6 has been shown to be involved in prostate cancer [64], and presence of MAST1 mutations has been detected in lung samples [65]. The expression of CYSLTR2 gene is a prognostic marker in colon cancer [66]. RPS6KA2 gene is a putative tumor suppressor gene in ovarian cancer [67], and FES is a known proto-oncogene [68]. BAX is an oncoprotein with known role in cancers [69], including lung cancer [70]. Mutations in the PIK3C2B gene were previously observed in lung and other tumors [71, 72]. There is emerging evidence of a role of RANBP9 gene in lung cancer [73]. The 67-kDA laminin receptor gene RPSA, while not present in DriverDBv2, is known to play a role in tumor growth [74, 75].

Among the putative driver genes discovered by QuaDMutEx in the BRCA samples, nine were mutated in four or fewer of the 771 patients. Two among the genes that were mutated in more than four patients were not present in the DriverDBv2 database: NBPF1 and KIAA1310. However, NBPF1 has recently been identified as tumor suppressor gene [76]. KIAA1310 (KANSL3) is a member of KANSL family which plays a role in cell cycle and reduction of its function is associated with cancer [77]. Of the rarely mutated genes, only TSNARE1 gene is likely to be a false positive. CASP8AP2 gene has been previously linked to cancer [78, 79]. No direct role in oncogenesis for ADCY1 gene has been reported, however it has been found downregulated in osteosarcomas [80]. PITX2 is a recurrence marker in breast cancer [81]. PSG11 gene has been shown to be correlated with survival in ovarian cancer [82]. Ankyrin repeat proteins, though not ANKRD34B specifically, have been previously reported as promoting cancer development [83]. KRT14 gene dysregulation was recently linked with breast cancer metastases [84]. MSI1 is putative therapeutic target in colon cancer [85]. TWISTNB is a component of the RNA polymerase I complex, and while TWISTNB gene has not been previously linked to cancer, mutations in polymerase subunits, cofactors, and mediators are known factors in malignancy [86]. Together, these results confirm that QuaDMutEx is effective in identifying cancer driver mutations even if they are rare in the analyzed patient group.

### Comparison with gene expression-based driver discovery

In addition to methods that use only genomic mutation data, we also compared QuaDMutEx to DriverNet, a method that uses a biological network and gene expression data in addition to mutation data. We used four genomic-transcriptomic datasets that are provided with the DriverNet tool: triple negative breast cancer (eTNB), glioblastoma multiforme (eGBM), high-grade serous ovarian cancer (eHGS), and METABRIC breast cancer (eMTB) datasets. The summaries of the datasets are provided in Table 5.

**Table 5:** Summary of genomic-transcriptomic datasets used in comparison with DriverNet

Dataset	Samples (n)	Genes (p)	Mutations
eTNB	94	4594	6007
eGBM	120	3747	8141
eHGS	316	13278	22897
eMTB	696	13076	51255

DriverNet was executed using default parameters on the full information contained in the dataset, that is, the genomic, transcriptomic, and biological network information. The solution gene sets include all genes found by DriverNet to be statistically significant at the 0.05 *p*-value threshold. QuaDMutEx was executed using only the genomic data describing presence or absence of a mutation in a given gene in a given patient. We used the default value of *k*=1, and set the value of *C* to 1.5, with the exception of the smallest dataset, eTNB, for which we used *C*=1. We compared the putative cancer driver gene sets discovered by the two tools using coverage, excess coverage, and the Dendrix score, as described above.

For the eGBM dataset, QuaDMutEx shows much higher coverage and much lower excess coverage (see Table 6). For the other three datasets, QuaDMutEx shows much lower excess coverage than DriverNet, at the cost of a moderate decrease in coverage. These results reflect the fact that DriverNet is not designed to take mutual exclusivity of genes into consideration. On the other hand, DriverNet return many more genes than QuaDMutEx. A single run of QuaDMutEx is designed to return a single set of genes with low excess coverage, and does not include all putative driver genes - these can be detected with another run of QuaDMutEx.

**Table 6:** Comparison between QuaDMutEx and DriverNet

Method	Genes	Coverage	Excess	Dendrix
			coverage	score
eTNB: Triple negative breast cancer				
DriverNet	64	0.6809	0.4688	18
QuaDMutEx (C=1)	16	0.8315	0.0270	*72*
eGBM: Glioblastoma multiforme				
DriverNet	19	0.9412	0.8839	-183
QuaDMutEx (C=1.5)	6	0.8067	0.0938	*87*
eHGS: high-grade serous ovarian cancer				
DriverNet	77	0.9430	0.6946	-110
QuaDMutEx (C=1.5)	14	0.8734	0	*276*
eMTB: METABRIC breast cancer				
DriverNet	92	0.4670	0.7785	-1151
QuaDMutEx (C=1.5)	18	0.4071	0.0876	*250*

Highest result for each dataset indicated in italics

To provide a comparison that does not involve mutual exclusivity, we used the COSMIC database of mutations in cancer, and we introduced iterated QuaDMutEx, which increases the number of genes found by QuaDMutEx to the numbers similar to DriverNet. We performed four executions of QuaDMutEx, after each run removing the genes discovered so far from the dataset, so that they do not prevent discovery of additional genes that are not mutually exclusive with previously discovered ones. We then pooled the four high-exclusivity gene sets into a single high-coverage set. Since mutual exclusivity can be expected only for a set of functionally-related genes, for example genes from a single cancer-related pathway, a single call to QuaDMutEx corresponds to a single-pathway query, and calling QuaDMutEx iteratively corresponds to a multi-pathway query, facilitating comparison with DriverNet which does not have a single-pathway focus.

**Fig. 6:**
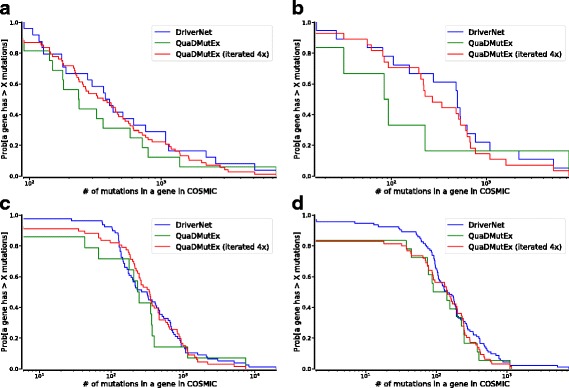
Complementary cumulative distribution function plots for QuaDMutEx, iterated QuaDMutEx, and DriverNet, for eTNB (**a**), eGBM (**b**), eHGS (**c**), and eMTB (**d**) datasets

To measure the quality of solutions returned by DriverNet and iterated QuaDMutEx in a way independent of any mutual exclusivity of gene mutations, we compared the numbers of COSMIC occurrences of mutations in genes returned by DriverNet with occurrence numbers for QuaDMutEx gene sets. Specifically, for each gene in a discovered gene set, we queried COSMIC for the number of observed mutations in that gene. We then plotted a complementary cumulative distribution function (CCDF) over the numbers over the whole gene set. For example, for the eHGS dataset, for both QuaDMutEx and DriverNet, the CCDF value at 1000 is approximately 0.14, indicating that for both methods, 14% of the genes in the solution set have more than 1000 mutation each in COSMIC, while for 86% of genes in the solution set a COSMIC query for the gene results in at most 1000 mutations. The results in Fig. 6 indicate that iterated QuaDMutEx and DriverNet perform similarly on eTNB and eGBM datasets, and on eHGS and eMTB both perform similarly for majority of the mutation counts range, with DriverNet having an edge at the numbers below that threshold.

**Fig. 7:**
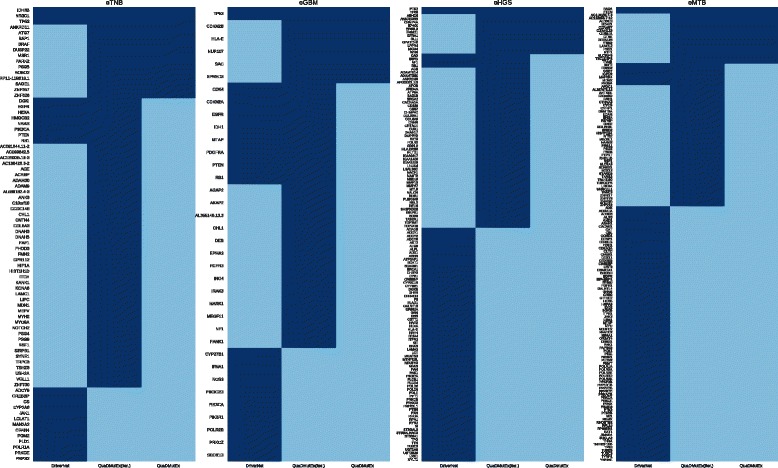
Comparison of putative cancer driver gene sets returned by QuaDMutEx, iterated QuaDMutEx, and DriverNet. Genes found by a tool are in dark blue

Genes returned by QuaDMutEx are to large extent different than those returned by DriverNet (see Fig. 7), showing that the expression-based approach used in DriverNet and the mutation-only approach used in QuaDMutEx are complementary. We validated the genes discovered by QuaDMutEx (Table 7) in DriverDB2, a database of genes previously discovered as cancer drivers. For eTNB and eGBM datasets, all the genes discovered by QuaDMutEx are present in DriverDB2 database. In eHGS dataset, only ANKRD36B was not found in DriverDB2. However, ANKRD36B gene was identified in rare germline copy number variations in renal clear cell carcinoma [87], and also correlates with cellular sensitivity to chemotherapeutic agents [88]. In eMTB dataset, TRA@ gene is not present in DriverDB2, but it has been previously found to be linked to breast cancer [89]. TRA@ os also one of the genes that were discovered both by DriverNet and by QuaDMutEx. TBC1D3P2 is recurrently mutated in meningioma cell lines [90] and is a pseudogene for TBC1D3, a known oncogene [91]. There is no information available about AC116655.7-12 and AC116165.7-3, and at this point we classify both as false positives.

**Table 7:** Putative driver gene sets discovered by QuaDMutEx

Putative driver genes	Estimated
discovered by QuaDMutEx	*p*-value
eTNB: Triple negative breast cancer	
**TP53** (35) **PARK2** (6) **ROBO2 DUSP22** (4) **SAGE1 ANKRD11 NR3C1** (3) **BAP1 BRAF ATG7** (2) **ZNF257 IDH3B ZNF826 RP11-119B16.1 PSG5 MSR1** (2)	0.001
eGBM: Glioblastoma multiforme	
**CDKN2B** (52) **TP53** (38) **NUP107** (9) **HLA-E SAC SPRED3**	0.001
eHGS: high-grade serous ovarian cancer	
**TP53** (249) **GLI1** (3) **ABHD6 CHMP4A EP400 EPS8L3 FRMD1** (2) **GPATCH8 MCM4 GFRA1 LPPR4 PTK2 WRN** ANKRD36B (2)	0.001
eMTB: METABRIC breast cancer	
**C17orf37(MIEN1)** (82) **BAG4** (52) **CLNS1A** (37) **PSG1** (24) **C20orf133(MACROD2)** (19) **BCAS1** (17) **PTEN**(16) **RTF1** **ALOXE3** (7) TRA@ AC116165.7-3 (6) TBC1D3P2 (5) **CTSK** AC116655.7-12 **LANCL2** (4) **ITSN2** (3) **DEFB126** (3) **SLC35F3** (2)	0.001

For each gene, in parentheses, we provide the number of patients in the dataset that harbored a mutation in that gene. Genes in bold are present in the DriverDBv2 [7] database of previously discovered cancer drivers

## Conclusions

Superior ability to improve on both coverage and excess coverage of the detected driver gen sets on datasets from different types of cancer shows that QuaDMutEx is a tool that should be part of a state-of-the-art toolbox in the driver gene discovery pipeline. It can help detect low-frequency driver genes that can be missed by existing methods.
